# Evaluation of the opinions of midwifery students on adolescent pregnancy: A phenomenological qualitative study in eastern Turkey

**DOI:** 10.18332/ejm/138593

**Published:** 2021-10-25

**Authors:** Sibel Öztürk

**Affiliations:** 1 Department of Midwifery, Faculty of Health Sciences, Atatürk University, Erzurum, Turkey

**Keywords:** adolescent, pregnancy, midwifery, midwifery education, midwifery student

## Abstract

**INTRODUCTION:**

Adolescent pregnancy is a major health problem that is significantly associated with adverse impacts on the health of both teenage mothers and their newborns. This study was conducted to evaluate the opinions of midwifery students on adolescent pregnancy.

**METHODS:**

The participants were selected using a purposeful sampling method. Thirty-seven students studying at the final year of the midwifery department were interviewed in the study in Erzurum, Turkey, in 2018. The interviews were analyzed by the phenomenological qualitative method.

**RESULTS:**

Students thought that adolescent pregnancy was a social problem in addition to its maternal and fetal risks. The students indicated that adolescent pregnancy was perceived as normal in a family in which cultural values were important (especially in those families that live in the Eastern and South-Eastern regions). Only one student attracted attention to the effect of the media in this respect. The students stated that adolescent pregnancy was a social problem, and a multidisciplinary approach was required for its solution.

**CONCLUSIONS:**

Our qualitative study provides insight into the role of adolescent pregnancy in Turkey. Participants expressed the idea that increasing the education level of girls and raising awareness among mothers may be an effective solution to the problem. The students were aware that adolescent pregnancy was an important social problem.

## INTRODUCTION

Adolescent pregnancy is a major health problem that is significantly associated with adverse impacts on the health of both teenage mothers and their newborns^1,2^. Various health problems are associated with the negative outcomes of pregnancy during adolescence^3^, including anemia, preeclampsia-eclampsia, human immunodeficiency virus and other sexually transmitted infections, postpartum hemorrhage, and mental disorders (e.g. depression). Perinatal outcomes are preterm birth, intrauterine growth restriction, low birth weight, and admissions to intensive care units^4,5^. Adverse birth outcomes associated with adolescent pregnancies not only affect the health of mothers and infants but also influence familial and societal aspects^6,7^. Adolescent pregnancy also affects women’s abilities to pursue employment and educational opportunities, leading to an increased rate of subsequent poverty for mothers and higher risk for behavioral problems, and lower educational attainment for children^4,8^. Marriages during the adolescence period reduce the self-confidence of adolescents and make it hard for them to develop a unique identity. The failure to cope with postnatal difficulties and low self-respect are also observed among adolescent mothers^9^.

Adolescent marriage and pregnancy are a global problem that is observed worldwide^3^. Nevertheless, its distribution around the world varies. This difference originates from various social, cultural, and economic factors such as traditional attitudes, religious beliefs, family structure, education level, economic situation, and access to family planning services^10^. The regions where this problem is most prevalent are South Asia, North Africa, the Middle East, Latin America, and West African countries^10^.

Of adolescent women, 5% gave birth in Turkey. At this age, 3% of women had given live birth, while 1% were pregnant with their first child at the time of the research. In 2008, the adolescent pregnancy rate was 6% according to the Turkish Demographic and Health Survey (TDHS), which appeared to decrease to 4% according to the 2018 TDHS results^11^. Although this rate has decreased over time, Turkey receives a lot of migration from undeveloped countries (Afghanistan, Syria, South Asia, many countries in Africa) due to its geographical location^12,13^. Therefore, adolescent pregnancy is still significant in Turkey.

The practitioner, instructor, manager, and researcher roles of nurses and midwives concerning their professions are also important in adolescent pregnancy, which is a social problem. Midwives and nurses have significant duties, especially in preventing adolescent pregnancies^10,14^. While the rate of adolescent pregnancies in Turkey is gradually decreasing, they are still present^11^. The rate of adolescent pregnancy is higher in the Eastern and South-Eastern regions of Turkey^11^. International migrations that have increased in recent years also affect this situation. The approach of midwives, who take a caregiver role and perform follow-up in the prenatal-postnatal periods and during pregnancy, toward pregnant adolescents is also important. Final-year students are soon assigned as midwives in the clinic or in the field. Therefore, it is important to evaluate the attitudes of final-year students when they encounter a pregnant adolescent before their graduation. The study was conducted to assess the opinions of midwifery students on adolescent pregnancy.

## METHODS

This research is a qualitative phenomenological study. Phenomenology is an approach that ensures thinking about opinions and human experiences, which constitute the basis of qualitative research. It is important to determine the attitudes and thoughts of students who will graduate and practice their profession in the near future when they come across pregnant adolescents.

### Setting and participants

This study is based on interviews regarding midwifery students’ thoughts about and approaches toward adolescent pregnancy. The participants were selected using a purposeful sampling method. Thirty-seven students studying in the final 4th year of the Department of Midwifery at the Faculty of Health Sciences were interviewed in Erzurum. The reason for interviewing final-year students is that they are at the final stage of their education and they have more professional experience and knowledge compared to students in the earlier years. Only Turkish students were included in the scope of the study. All Turkish students in the 4th year participated in this study. The participation rate was 94.9%; there were a total of 40 students in the class, and one student did not wish to participate in the research. Two students, who were of foreign nationality, were not included in the interviews.

### Data collection

Data for the qualitative phase of the study were collected during January – February 2018. In-depth, individual, face-to-face, semi-structured interviews were held with 37 midwifery students. The interviews were held in an empty class where it was possible to establish comfortable communication with students. The interviews were held when students were most ready for the interview. The interview was held by sitting face-to-face at the same level as the students and guided with question forms through active listening. The semi-structured interviews were audio recorded. The interviews lasted 25 minutes on average (range: 15–45 minutes).

### Data collection tool

A semi-structured interview form was used with the students, which consisted of questions that evaluated the student’s opinions about and approaches toward adolescent pregnancy. The questions (e.g. ‘What do you think about pregnant adolescents?’, ‘Is adolescent pregnancy a social problem?’, ‘Does culture have an effect on adolescent pregnancy?’) were derived from a literature review on the topic and based on professional knowledge and experience^1,9,15,16^. The data collected were rich in content since all the students spoke freely and extensively about their experiences. The author undertook all the interviews. A pilot study was performed by interviewing three students to ensure the validity and reliability of the interview form. The necessary modifications were made in the interview form after the pilot study.

### Statistical analysis

The interviews were tape-recorded and transcribed verbatim for phenomenological analysis. The transcribed interviews were analyzed carefully by the author using systematic text condensation. First, the interviews were transcribed verbatim and re-read several times to obtain a global sense of the data. To analyze the data, and thus gain a deeper understanding, the text was divided into meaning units^15^. The next phase was to group meaning units related to each other into codes of meanings^17,18^. After coding all meaning units, interrelationship patterns of the phenomenon were identified, and constituents were defined. All codes were reread to identify possibly missing meaning units^18^. The aim was to keep the analysis close to the original data with a minimum number of generalities or abstractions. Finally, the essence of the phenomenon was formulated^16,19^. One of the strategies proposed for the internal reliability of qualitative research is to receive an expert opinion from another researcher in the analysis of the data. This ensures that the results are not the researcher’s opinions and are based on the actual data obtained^20^. Therefore, the researcher received an expert opinion after creating themes and codes. Furthermore, the researcher provided direct quotations from the participants’ statements while interpreting the results to strengthen the validity of the study results. Since the phenomenological approach is discovery-oriented, the analysis was left open in order to let unexpected meanings emerge. During the process, a new comprehension of the phenomenon emerged. The analysis was performed in Turkish. The data analysis was finally directly translated into English, aiming to convey the original sense. The quotations were checked for accuracy by two translators who are native English speakers.

Permission for this study was obtained from the Ethics Committee of Atatürk University, Faculty of Health Sciences (14.12.2017). Importance was attached to the voluntary participation of the students. Furthermore, oral and written consent was obtained (the principle of informed consent) after explaining the study aim and the purposes for which the results obtained would be used to the students. It was explained to the students that information about them would not be disclosed to others, and the principle of confidentiality was complied with.

## RESULTS

### Participant characteristics

The mean age of the students participating in the study was 21.62 ± 0.72 years. Seventeen students lived in the Eastern and South-Eastern Region, four students lived in the Aegean Region, six students lived in the North Anatolia Region, two students lived in the Central Anatolia Region, and eight students lived in the Mediterranean Region. All of the students who participated in the study were 4th year students.

### Adolescent pregnancy is a social problem

All students stated that adolescent pregnancy was risky, and it was a social problem. Students thought that adolescents could not adapt to the role of motherhood as their physical and emotional development continued. The participants believed that leaving their education life half-finished would also affect raising the child adversely, and they would not be able to raise socially beneficial individuals:

*‘I think that adolescent pregnancy is a social problem. Even the physiological development of adolescents is not completed yet. I think adolescent mothers will not be able to raise their children efficiently since their level of education is low (generally at the primary school level).’* (Student 5)

*‘Yes. Children raised by mothers make up society.’* (Student 8)

*‘Yes, because adolescent pregnancy is risky. It means an unhealthy pregnancy, and an unhealthy child is equal to an unhealthy society.’* (Student 1, Student 9)

*‘Yes, because women and children raised by them determine the quality and future of society. An adolescent who becomes pregnant before completing personal development cannot bring well-educated children and adults to society. This increases social problems.’* (Student 15)

*‘Yes. It is an important problem for the future of the family and our country. Children raised by mothers make up society.’* (Student 32, Student 28)

### Adolescent pregnancy and business life

Most of the students thought that pregnant adolescents could not become successful in education and business life. The participants stated that incomplete education would prevent them from getting a profession and they would work in low-paying jobs. A few students expressed the idea that adolescent mothers could get a job and succeed only after raising their children since they became mothers at an early age:

*‘Pregnant adolescents may not succeed in their education and business life in subsequent years. Their motherhood is an obstacle to this. I think their rate of success in completing their unfinished education is also low.’* (Student 5)

*‘Motherhood is a very significant responsibility in human life, and it requires sparing time. Therefore, I do not think that they can become successful.’* (Student 1)

*‘No, because they will have the responsibility of pregnancy and the baby. Therefore, I do not think that they will become successful in education and business life.’* (Student 33)

*‘I don't know. Maybe they can start it all over since they raise their children in an early period. But maybe they will be worn-out since they have become mothers at an early age and undertaken significant responsibilities.’* (Student 22)

*‘Maybe they can succeed. Nothing is an obstacle to achieving our targets in life.’* (Student 32)

### Adolescent pregnancy and culture

All the students answered by considering that pregnancies occurred after marriage because marriage means a child, and a child means marriage in our country. Even this perception shows the effect of culture on students. Almost all students thought that culture affected marriage. They stated that adolescent marriage and pregnancy were present in societies deeply attached to their culture, which was considered natural. They stated that the rates of adolescent marriage were higher in rural areas. They thought that adolescent pregnancy was perceived as natural in families living in the regions of our country where cultural values were effective (especially in the Eastern and South-Eastern regions). Interestingly, only one student stated that the media had an encouraging effect:

*‘Culture has a significant effect on adolescent pregnancy. It is more common in regions where cultural values are effective.’* (Student 31)

*‘There is a cultural effect. I am a person raised in such a culture. The adolescent and the family have the perception that an individual cannot marry at an older age (20 years and above) if he/she does not marry at an early age. The age of 20 years and above is regarded as late for marriage. This thought normalizes an individual getting married and having children at an early age. It even causes social pressure on the individual.’* (Student 20)

*‘I believe that culture has a significant effect on adolescent pregnancy. Families who still think that girls should not go to school either marry their daughters at an early age or approve their wish.’* (Student 5)

*‘I think that culture has an effect since adolescent pregnancies are observed among those who live in rural areas.’* (Student 22)

*‘Yes, marriage and pregnancies are encouraged since adolescent marriages are perceived as normal in certain regions of our country.’* (Student 15)

*‘I think adolescent marriage and pregnancies occur as a result of culture. This is right. But I think that media are much more effective in today's adolescent pregnancies, and it encourages adolescents to marry. Boyfriend and girlfriend scenes in TV series and films in the media affect adolescents adversely. Also, certain families do not fulfill their responsibilities toward their children. And adolescents prefer to marry when they do not receive the attention they expect from the family. They seek the interest they do not receive from their parents in their spouses.’* (Student 35)

### Adolescence and motherhood

There were three opinions on adolescent motherhood. The majority believed that an adolescent definitely could not become a good and adequate mother and that the adolescent was still a child. The second group thought that it was not correct to generalize, and this would vary from person to person. The third group (only two students) thought that adolescents would become good mothers with social support. They emphasized that cultural features were important in adopting the role of motherhood. They also stated that motherhood was instinctive.

First opinion:

*‘Motherhood is not all about giving birth to the baby. It's a role that requires responsibility.’* (Student 17)

*‘I don't think so. Being a mother is a big responsibility.’* (Student 31)

*‘No, I don't think that they can bear such a responsibility as their personality development continues.’* (Student 26)

*‘No, I don't think that adolescents can take the right decisions about the problems related to their children when their own physical and emotional development is not fulfilled. They cannot fulfill such a responsibility effectively.’* (Student 7)

*‘No, she is still a child, she cannot fulfill the role of a mother.’* (Student 30)

Second opinion:

*‘Maybe. It varies depending on the cultural features and maternal attachment status of an adolescent.’* (Student 2)

*‘They can become good mothers by maturing and developing themselves over time.’* (Student 23)

*‘I cannot make such a generalization. This varies from person to person.’* (Student 33)

Third opinion:

*‘They can fulfill the role and responsibilities of motherhood if there are people who support adolescents and provide them with education on motherhood. They can become a very good mother with little effort since God created us to be mothers. Motherhood is instinctive.’* (Student 5)

*‘They can be. It depends on the way the adolescent is raised. They can become good mothers if they are raised by having them adopt the role of motherhood.’* (Student 12)

### Adolescent pregnancy and midwifery

The students believed that this subject should be addressed with a multidisciplinary approach. They thought that the problem would be solved by increasing the education level of girls and raising awareness among mothers. They stated that midwives’ planning individual or group education for mothers and adolescents would be effective in preventing adolescent pregnancy. They believed that this problem could be solved by focusing more on the education of girls as the country’s policy:

*‘Midwives must focus on their social responsibilities in these regions where cultural pressure is high, arrange individual and group education for mothers, and ensure their participation.’* (Student 2)

*‘This problem is eliminated by raising awareness in society. Everything can improve with the development of a society. And primarily mothers. We must further increase the rate of girls studying at high school and university. This is the rooted solution to the problem.’* (Student 34)

The students stated that it was necessary to approach pregnant adolescents without condemning them as a midwife. They stated that communication with pregnant adolescents was very important in planning treatment and care. It is necessary to provide them with psychological support, act without prejudice, and monitor them closely in terms of health risks. The students emphasized the importance of focusing on pregnant education sensitively, and monitoring the mother and infant in the postnatal period regarding maternal attachment:

*‘Counselling education and psychological support are very important in this group. They must be monitored, especially in terms of the mother–baby attachment.’* (Student 21)

*‘We must provide care and treatment when we encounter a pregnant adolescent without finding this strange. We must also use our educator role effectively since this group needs information and social support on pregnancy and the postpartum period.’* (Student 32)

*‘We must listen to them without prejudice and plan care by determining their problems and needs.’* (Student 36)

## DISCUSSION

Adolescent pregnancy remains a significant social, economic, and health issue worldwide^21,22^. Adolescent pregnancy and motherhood are also often associated with lower education level and expose adolescent mothers and their infants to a negative future^23^. Young people undergo a high level of hardship and deprivation and suffer from a high level of emotional and behavioral disturbance^21,24^. Adolescent pregnancy causes worse health than that of the general population. In several studies, adolescent mothers had higher levels of psychological distress than their childless adolescent peers^25-28^. Studies in the literature mainly focus on the physiological, psychological, and socioeconomic effects of adolescent pregnancy on the mother and fetus^1,2,4,5,8,21,24^. There is no study in the literature on the social and emotional development of children of adolescent mothers. Unlike the literature, the students attracted attention to the approach of an adolescent mother to her child, the social and emotional status of the child, and the contribution of the child to society. The students’ answers provided a different point-of-view on this problem.

The common public image of adolescent pregnancy as a serious social problem is underscored by its perceived destructive consequences believed to be the outcome of lower education level, welfare dependency, and low-paying jobs, as well as more significant health problems for such mothers and their children^11,21,22^. The students thought that pregnancy would affect the education and working life of adolescents adversely. The students’ responses in compliance with the literature showed that they were knowledgeable about the present situation and were aware of the problems related to their profession.

When the effect of culture on adolescent pregnancy was asked to the students, all the students perceived this as a pregnancy that occurred as a result of marriage and answered the question by thinking in this way. They did not even think that pregnancy could be extra-marital. Extra-marital sexual intercourse is not accepted in Turkish culture and belief (Islam religion). This approach of the students shows that they have adopted the cultural and religious rules. The students thought that culture affected adolescent marriage. They stated that adolescent marriage and pregnancy were more common in regions where cultural values were important (in the Eastern and South-Eastern regions). In Turkey, the rate of adolescent marriage is higher in the Eastern and South-Eastern regions. The results of the study conducted by Aydin and Akay^15^ also showed that the lack of education and cultural factors ranked first in adolescent marriages. The students’ answers showed that they analyzed the present situation well.

One student believed that media might encourage adolescent pregnancy. Nowadays, the media provides very rapid information flow to large masses in a short time. Especially, the visual media may convey negative messages most of the time. The media focus on women in terms of beauty and sexuality^29^. This perception created by the media may have encouraged adolescents in the process of growth and development toward sexual activity.

The fact that extra-marital sexual intercourse is not regarded as usual in Turkey may have also led adolescents toward marriage. The problem of adolescent pregnancy is addressed from its educational and cultural aspects in the literature. The fact that the student attracted attention to the impact of media on adolescents and addressed the subject from this aspect is quite interesting. There is no finding in the literature that supports this point-of-view of the student, but the matter may also be evaluated in this respect, considering the impact of media on the youth.

Students had three different thoughts about adolescent motherhood. The fact that most of the students did not support adolescent motherhood, although most of them lived in the Eastern and South-Eastern regions, where cultural values were effective, can be explained by the effect of education (the students who participated in the study are final-year students at the university). Furthermore, the fact that there are adolescent mothers in the area where the students live and that their mothers also became mothers at a young age may be effective in not supporting adolescent motherhood.

Students regard adolescent pregnancy as a social problem. Therefore, they think that a multidisciplinary approach that includes midwives is required to solve the problem. This approach of the students did not restrict their professional knowledge, skills, and responsibilities to the clinic. This point-of-view of the students shows that they are aware of the importance of their profession in solving social problems.

The students defined pregnant adolescents as a risk group and stated that it was necessary not to be prejudiced against them. Answers given by the students showed that their professional knowledge and responsibilities were wellestablished. This approach of the students is important for protecting and developing the health of the mother and child.

### Limitations

Phenomenology studies may not reveal precise and generalizable results in accordance with the nature of qualitative research. The most significant limitation of this study is that no generalization could be made. The interview was held on the pregnancies that occurred as a result of marriage. Extra-marital pregnancies were not part of the interview.

### Future research

There are many areas identified from this study for future research. In this study, the students answered the questions on pregnancy occurring as a result of marriage. Therefore, the approaches of midwifery students toward the pregnancies that occur as a result of extra-marital relations may be assessed. Moreover, future studies can be conducted on the children of adolescent mothers to assess their psychosocial development. One student attracted attention to the media in adolescent pregnancy. Therefore, the impact of the media on adolescent marriages can be investigated. This research can be guiding for studies that question the impact of the media on the sexual life of adolescents.

## CONCLUSIONS

All the students perceived adolescent pregnancy as occurring as a result of marriage and answered the questions accordingly. The subject may be evaluated separately by holding a separate interview for extra-marital pregnancies. The students addressed the problems related to adolescent pregnancy not only from maternal, fetal, and neonatal aspects but also in terms of society. The students believed that children raised by adolescents would not contribute to society positively. In line with this point-of-view, studies can be conducted on children born to adolescent mothers. Most of the students thought that adolescent pregnancy had a cultural effect. The students stated that the solution to this problem required a multidisciplinary approach. This shows that midwifery is important for detecting the existing problems effectively and correctly and finding realistic solutions for them with education at the university level.

## Data Availability

The data supporting this research are available from the author on reasonable request.
